# Childhood cognitive control as a predictor of long‐term clinical and functional outcomes in Tourette syndrome

**DOI:** 10.1111/jcpp.70155

**Published:** 2026-04-21

**Authors:** Kathryn E. Barber, Emily J. Ricketts, Douglas W. Woods, John Piacentini, Flint M. Espil, Matthew Specht, Shannon M. Bennett, John T. Walkup, Susanna Chang, Alan L. Peterson, Lawrence Scahill, Sabine Wilhelm, Joseph F. McGuire

**Affiliations:** ^1^ Department of Psychology Marquette University Milwaukee Wisconsin USA; ^2^ Department of Psychiatry and Biobehavioral Sciences University of California, Los Angeles Los Angeles California USA; ^3^ Department of Psychology Loyola University Chicago Chicago Illinois USA; ^4^ Department of Psychiatry and Behavioral Sciences Stanford University School of Medicine Stanford California USA; ^5^ Department of Psychiatry Weill Cornell Medicine New York New York USA; ^6^ Ann & Robert H. Lurie Children's Hospital of Chicago Chicago Illinois USA; ^7^ Northwestern University Feinberg School of Medicine Chicago Illinois USA; ^8^ Department of Psychiatry and Behavioral Sciences University of Texas Health Science Center at San Antonio San Antonio Texas USA; ^9^ Marcus Autism Center Emory University School of Medicine Atlanta Georgia USA; ^10^ Massachusetts General Hospital/Harvard Medical School Boston Massachusetts USA; ^11^ Department of Psychiatry and Behavioral Sciences Johns Hopkins University School of Medicine Baltimore Maryland USA

**Keywords:** Executive function, inhibition, working memory, set‐shifting, longitudinal, neurocognitive

## Abstract

**Background:**

Tourette syndrome (TS) is a childhood‐onset neuropsychiatric condition characterized by motor and vocal tics. Many individuals with TS continue to experience tics and functional difficulties into adulthood, yet the factors influencing these long‐term trajectories remain poorly understood. Cognitive control processes, implicated in the etiology and treatment of TS, may serve as indicators of later clinical and functional outcomes.

**Methods:**

This study tested whether childhood cognitive control predicted outcomes in early adulthood. Participants were 80 individuals with TS (*M*_age_ = 22.8 years, *SD* = 2.7) who had entered a randomized clinical trial of behavioral therapy as children and completed a follow‐up evaluation an average of 11.7 years (*SD* = 1.3) later. Childhood assessments measured tic severity and neurocognitive domains, including processing speed, inhibition, set‐shifting, and working memory. Follow‐up assessments evaluated clinical, functional, and quality of life outcomes.

**Results:**

Results showed that greater inhibition, set‐shifting, and processing speed in childhood predicted lower tic severity and impairment, greater odds of tic remission, and higher quality of life in early adulthood. Greater working memory and response flexibility predicted higher educational attainment and income. These relationships remained significant after accounting for treatment conditions and comorbid attention‐deficit/hyperactivity disorder and obsessive‐compulsive disorder.

**Conclusions:**

Findings highlight childhood cognitive control as an important predictor of clinical and functional outcomes in TS and a viable target for intervention.

## Introduction

Tourette's disorder and persistent (chronic) tic disorder – collectively referred to as Tourette syndrome (TS) – are childhood‐onset neuropsychiatric conditions characterized by motor and/or vocal tics that are present for longer than 1 year (American Psychiatric Association, 2022). TS affects about 1% of the population, with symptoms typically emerging in early childhood and peaking in severity in early adolescence (Bloch et al., 2006; Hirschtritt et al., 2015). Alongside tics, 86% of individuals with TS experience comorbid psychiatric conditions that include attention‐deficit/hyperactivity disorder (ADHD), obsessive‐compulsive disorder (OCD), or anxiety disorders (Hirschtritt et al., 2015; Wolicki et al., 2019). Together, TS and accompanying psychiatric comorbidities cause significant distress, executive dysfunction (i.e., reduced cognitive control; Morand‐Beaulieu, Stark, & Murphy, 2025), and functional impairment (Cloes et al., 2017; Storch et al., 2007) – all of which contribute to a reduced quality of life (Eapen, Cavanna, & Robertson, 2016).

For over 60% of youth with TS, tics continue into late adolescence and early adulthood (Bloch & Leckman, 2009; Reagan, Myers, & McGuire, 2022). Although evidence‐based treatments exist to reduce tic severity (e.g., behavior therapy, pharmacotherapy; Pringsheim et al., 2019), these interventions infrequently produce tic remission. Importantly, even for those individuals who experience tic remission, functional difficulties can persist. Many adults with TS report ongoing challenges across academic, occupational, and social domains, with some attributing school or work disruptions (e.g., dropping out, avoiding career advancement) to their childhood history of tics (Coffey et al., 2004; Conelea et al., 2013; Pérez‐Vigil et al., 2018; Shady, Broder, Staley, Furer, & Brezden Papadopolos, 1995). Indeed, population‐based studies show that individuals with TS are less likely to complete secondary or postsecondary education, are more likely to be underemployed, and report lower income levels than unaffected counterparts (Pérez‐Vigil et al., 2018; Yang et al., 2016). Overall, these findings highlight the enduring challenges associated with TS and underscore the importance of identifying factors that influence long‐term outcomes.

Although the lasting clinical and functional consequences of TS are well documented, few studies have examined predictors of these trajectories using prospective designs (Reagan et al., 2022; Ricketts et al., 2022), and the limited available evidence is inconsistent. For instance, some reports suggest that comorbid ADHD and OCD in childhood are associated with reduced likelihood of tic remission in adulthood (Byler et al., 2014; Groth, Debes, & Skov, 2017; Lowe, Capriotti, & McBurnett, 2019), whereas other studies have not supported this relationship (Bloch et al., 2006; Groth, Skov, Lange, & Debes, 2019). Meanwhile, no research has directly examined childhood predictors of later functioning or quality of life in TS. Addressing this gap is essential to clarify sources of variability in both clinical course and functioning, which could provide mechanistic insights and inform interventions that promote more favorable long‐term outcomes.

One candidate factor that is implicated in TS and its common comorbidities (e.g., ADHD, OCD) is cognitive control (Sadozai et al., 2024; Snyder, Kaiser, Warren, & Heller, 2015; Willcutt, Doyle, Nigg, Faraone, & Pennington, 2005). Cognitive control refers to higher‐order mechanisms that regulate mental activity and behavior to enable purposeful, goal‐directed action, with key processes including inhibition, set‐shifting, attention maintenance, processing speed, and working memory (Ambrosini, Arbula, Rossato, Pacella, & Vallesi, 2019; Banich, 2009; Funahashi & Andreau, 2013). Evidence suggests that individuals with TS show impairments across these domains relative to controls (Cavanna et al., 2020; Morand‐Beaulieu et al., 2025), with some deficits more pronounced among those with comorbid conditions such as ADHD or OCD (de Groot, Yeates, Baker, & Bornstein, 1997; Morand‐Beaulieu et al., 2017; Müller et al., 2003). In particular, studies comparing youth with TS with and without ADHD have reported poorer performance among those with co‐occurring ADHD on cognitive control tasks, such as the Stroop, Go/No‐Go, and stop‐signal paradigms (Chang et al., 2018; Morand‐Beaulieu et al., 2017; Sukhodolsky, Landeros‐Weisenberger, Scahill, Leckman, & Schultz, 2010).

Cognitive control has also been recognized as a factor influencing tic severity reductions in behavioral interventions for TS. However, findings in adults are mixed. In one report, pretreatment inhibitory control predicted a positive response to behavior therapy (Deckersbach, Rauch, Buhlmann, & Wilhelm, 2006), while another report found no such association (Abramovitch et al., 2017). Notably, among children with TS, stronger working memory and inhibition predicted clinical improvement across both behavior therapy and a psychoeducation and supportive therapy control condition (Chang et al., 2018), and stronger baseline inhibition and set‐shifting predicted greater tic reduction following behavior therapy in another trial (McGuire et al., 2021b). Taken together, findings suggest developmental differences exist – such that in childhood, cognitive control may play a greater role in supporting the self‐regulation required for suppression‐based interventions (Essoe, Ramsey, Singer, Grados, & McGuire, 2021).

Beyond treatment response, childhood cognitive control may confer advantages that shape later functional outcomes and quality of life. For instance, greater cognitive control (e.g., behavioral regulation) in diverse settings and/or across time can contribute to academic attainment, occupational success, and overall functioning. Indeed, longitudinal research in both ADHD and typically developing samples has found a relationship between childhood cognitive control and future academic, occupational, and psychosocial outcomes (Ahmed, Tang, Waters, & Davis‐Kean, 2019; Barkley & Fischer, 2011; Cheng & Furnham, 2012; Miller, Nevado‐Montenegro, & Hinshaw, 2012; Stucke & Doebel, 2024). Thus, a similar prospective relationship may exist in TS, with difficulties in late adolescence and early adulthood potentially reflecting lasting neurocognitive sequelae (Morand‐Beaulieu et al., 2025). Yet, no studies have directly examined long‐term associations between these factors.

In response to this critical literature gap, we examined the predictive role of childhood cognitive control among youth with TS who were prospectively followed into late adolescence and/or early adulthood (approximately 11 years after an initial evaluation). Here, we examined whether childhood cognitive control predicted long‐term outcomes, focusing on (1) tic severity and impairment, (2) functional outcomes (i.e., educational attainment, employment, income), and (3) quality of life.

## Methods

### Participants

Data for this study were collected as part of a long‐term follow‐up evaluation of a three‐site randomized control trial (RCT) examining behavior therapy for TS (Espil et al., 2022; Piacentini et al., 2010). The initial RCT enrolled 126 youth with TS, and 80 individuals (Baseline *M*_age_ = 11.5 years, *SD* = 2.4; Follow‐up *M*_age_ = 22.8 years, *SD* = 2.7) completed the long‐term follow‐up evaluation an average of 11.7 years (*SD* = 1.3; range = 8.1–13.7) later. Of these participants, 38 had originally been assigned to behavior therapy and 42 to a psychoeducation and supportive therapy control condition. There were no differences in baseline demographic or clinical characteristics between those who completed and did not complete the follow‐up assessment (see Espil et al., 2022). Most participants reported their sex as male (75%) and gender as male (75%), were White (86%), and attended partial or more college (66%). See Table 1 for full demographic information on the sample at follow‐up.

**Table 1:** Sample demographics

Characteristic		*N*	%
Sex assigned at birth	Male	60	75.0
	Female	20	25.0
Gender	Male	60	75.0
	Female	18	22.5
	Transgender/nonbinary	1	1.3
	Not reported	1	1.3
Race	White	69	86.3
	Black or African American	1	1.3
	American Indian or Alaska Native	0	0.0
	Asian	4	5.0
	Native Hawaiian or Other Pacific Islander	0	0.0
	Biracial	4	5.0
	Other	1	1.3
	Not reported	1	1.3
Education level	Less than 7 years of schooling	0	0.0
	Junior high school	0	0.0
	Partial high school	1	1.3
	High school graduate	12	15.0
	Technical college	4	5.0
	Partial college	28	35.0
	College graduate	24	30.0
	Professional degree	1	1.3
	Not reported	10	12.5
Employment	Full‐time employment	24	30.0
	Full‐time student	18	22.5
	Part‐time employment	16	20.0
	Part‐time student	1	1.3
	Unemployed	8	10.0
	Not reported	13	16.3
Income level	$14,999 or less	23	28.8
	$15,000–$34,999	19	23.8
	$35,000–$49,999	13	16.3
	$50,000–$74,999	3	3.8
	$75,000–$99,999	8	10.0
	$100,000–$149,999	0	0.0
	$150,000 or more	1	1.3
	Not reported	13	16.3

In the interim between the RCT and the follow‐up evaluation, 27 participants (34%) reported using tic‐influencing medication (e.g., α‐agonists, antipsychotics) at some point, and 8 (10%) were taking such medication at the time of reassessment. Forty‐seven participants (59%) received some form of mental health treatment during this period, although only 5 (6%) reported that this therapy specifically targeted tics, and 2 (3%) reported receiving behavior therapy for tics. Overall, 31 participants (39%) received an evidence‐based treatment for tics (tic‐influencing medication and/or behavior therapy) during the follow‐up period (Espil et al., 2022).

Baseline data from the RCT on parental education and occupation (classified as highest level attained between parents in two‐parent homes or by primary caregiver in single‐parent homes) indicated that 84% of participants had a parent with a college degree or higher (13% technical school or some college, 3% high school only). For occupation, 81% were classified as professional, 9% technician/skilled laborer, 5% craftsperson/artist, and 4% laborer/homemaker/clerical. These variables are reported descriptively to provide socioeconomic context for the sample at baseline.

### Measures

#### Anxiety Disorders Interview Schedule for DSM‐IV – Parent Version (ADIS‐IV‐P)

The ADIS‐IV‐P is a semi‐structured interview used to diagnose childhood psychiatric conditions (Silverman & Albano, 1996). The ADIS‐IV‐P has shown good psychometric properties (Jarrett, Wolff, & Ollendick, 2007; Lyneham, Abbott, & Rapee, 2007; Silverman, Saavedra, & Pina, 2001; Wood, Piacentini, Bergman, McCracken, & Barrios, 2002).

#### Stroop Color‐Word Task

The Stroop Task assesses processing speed, inhibition, and set‐shifting (Golden, Freshwater, & Golden, 2002). The task includes three consecutive trials: Color Naming (naming colors of solid squares), Word Reading (reading color words in black ink), and Color‐Word (naming ink color of incongruent color words, e.g. the word ‘red’ printed in blue ink). Trial completion times were converted to T‐scores. Higher Color and Word scores indicate faster processing speed. The Color‐Word Interference score is based on Color‐Word performance adjusted for Color and Word speed, with higher scores indicating greater interference (i.e., poorer inhibitory control and set‐shifting). This task has demonstrated good reliability and validity as a measure of cognitive control in both clinical and nonclinical populations (Scarpina & Tagini, 2017).

#### Stop Signal Task (SST)

The SST measures motor inhibitory control (Logan, 1994; Verbruggen & Logan, 2008). On Go trials, participants respond to an X or O with a key press. On Stop trials, a stop signal (red background) appears shortly after the X or O stimuli, indicating that participants should withhold the previously learned response. Stop signal reaction time (SSRT) is an index of the speed of the inhibition process, and Stop trial accuracy captures inhibitory success. The SST has demonstrated good reliability and construct validity as a measure of inhibitory control (Verbruggen & Logan, 2008; Verbruggen & McLaren, 2018).

#### Change Signal Task (CST)

The version of the SST used in this study also included CST conditions, which assess both inhibition and response flexibility. On Change trials, a change signal (blue background) appears shortly after the X or O, signaling to participants that they should inhibit the previously learned key press and instead press an alternate key. Change signal reaction time (CSRT) reflects the speed of both inhibition and execution of the alternate response, while Change trial accuracy indicates successful execution of the alternate response. The CST has been widely used to assess inhibitory control and response flexibility and has shown acceptable reliability in prior research (Boecker, Gauggel, & Drueke, 2013).

#### Auditory Consonant Trigrams (ACT)

The ACT measures verbal working memory and divided attention (Paniak, Miller, Murphy, Andrews, & Flynn, 1997; Stuss, Stethem, & Poirier, 1987). Participants are verbally presented with three consonants, then complete an interference task (counting backwards) for 3, 9, or 18 s. Participants are then asked to repeat the three consonants. Performance was indexed by the total number of consonants correctly recalled, with scores reflecting verbal working memory capacity under interference and divided attention. The ACT has demonstrated acceptable reliability as a measure of working memory and divided attention (Shura, Rowland, & Miskey, 2016; Strauss, Sherman, & Spreen, 2006).

#### Yale Global Tic Severity Scale (YGTSS)

The YGTSS is a clinician‐administered measure of tic severity and impairment (Leckman et al., 1989). The severity for motor and phonic tics is rated across five dimensions (number, frequency, intensity, complexity, and interference) and summed to produce a Total Tic Severity score (range: 0–50). A YGTSS Total Tic Score < 14 has been used in previous research to indicate tic remission (Espil et al., 2022; McGuire et al., 2021a; Ricketts et al., 2018). Tic‐related impairment (range: 0–50) is rated on a single dimension reflecting the extent to which tics interfere with daily functioning across domains, such as social relationships, academic or occupational performance, and family functioning. Ratings are anchored by clinical descriptors and range from no impairment (0) to severe impairment (50). The YGTSS has demonstrated strong psychometric properties (Leckman et al., 1989; McGuire et al., 2018; Storch et al., 2005).

#### Wechsler Abbreviated Scale of Intelligence (WASI)

The WASI is a brief, nationally standardized measure derived from the larger Wechsler intelligence scales (Wechsler Intelligence Scale for Children – Fourth Edition [WISC–IV] and Wechsler Adult Intelligence Scale – Third Edition [WAIS–III]) (Wechsler, 1999). The WASI yields a reliable estimate of Full Scale Intelligence Quotient (FSIQ), providing a measure of overall cognitive ability.

#### Demographics and Medical History

Participants reported information regarding demographics and medical history, including highest level of education attained, current occupation (employed, student, or unemployed), full‐ versus part‐time status if employed/student, job‐seeking status if unemployed, and income level.

#### Gilles de la Tourette Syndrome‐Quality of Life Scale (GTS‐QOL)

The GTS‐QOL is a 27‐item self‐report measure of TS‐specific health‐related quality of life (Cavanna et al., 2008). Items cover psychological, physical, obsessive‐compulsive, and cognitive domains, rated from 0 (no problem) to 4 (extreme problem). Total scores are normalized to a 0–100 scale, with higher scores indicating poorer quality of life. This measure has shown good psychometric properties (Cavanna, David, Orth, & Robertson, 2012), and internal consistency in this sample was high (Cronbach's alpha = .95).

#### Quality of Life Inventory (QOLI)

The QOLI is a 32‐item self‐report measure of quality of life across several domains (e.g., health, self‐esteem, work, friends, leisure, personal goals) (Frisch, 1994). Respondents rate each domain for importance (0 = not important to 2 = extremely important) and satisfaction (−3 = very dissatisfied to +3 = very satisfied). Weighted domain scores (importance × satisfaction) are averaged across important domains to yield an overall T‐score (Frisch, 2014). The QOLI has demonstrated good predictive validity and sensitivity to change (Frisch et al., 2005). Internal consistency of the QOLI in this sample was high (Cronbach's alpha = .88).

### Procedures

All procedures for the long‐term follow‐up study were approved by the University of California, Los Angeles Institutional Review Board (IRB#15‐001512). In the original RCT, parents or legal guardians provided written informed consent and children provided assent prior to participation. For the follow‐up study, written informed consent was obtained from all adult participants, and assent with permission from a parent or legal guardian was obtained for participants under 18 years of age.

At baseline, independent evaluators (IEs) administered interviews assessing tic severity (YGTSS), comorbid psychiatric diagnoses (ADIS‐IV‐P), and IQ (WASI), and research assistants administered tests of cognitive control (Stroop, SST, CST, ACT). Participants were randomized to receive either eight sessions of behavior therapy or psychoeducation and supportive therapy over 10 weeks (see Piacentini et al., 2010 for details). Clinical and cognitive control measures were re‐administered by assessors (IEs, research assistants) masked to treatment assignment at the post‐treatment assessment timepoint. IEs were trained to reliability prior to data collection and received ongoing supervision throughout the trial. Reliability of neurocognitive task administration was established through review and scoring of videotaped administrations by senior study personnel at each site.

Participants were recontacted to complete a long‐term follow‐up evaluation that occurred between March 2014 and January 2019, an average of 11.7 years (*SD* = 1.3) following the initial study treatment (see Espil et al., 2022 for details). IEs masked to original treatment condition conducted these assessments either in‐person or via videoconferencing. The IEs administered the YGTSS, and participants completed self‐report forms (demographics/history questionnaire, GTS‐QOL, and QOLI).

### Data analysis

Analyses tested whether childhood cognitive control performance, assessed at post‐treatment of the RCT, predicted long‐term follow‐up outcomes. When post‐treatment cognitive control scores were missing, values from baseline were carried forward (see Table 2). This conservative approach was selected due to limited information on reliable imputation methods for neurocognitive tests and evidence that behavior therapy for TS has little effect on neurocognitive functioning (Chang et al., 2018). Consistent with this, paired‐samples *t*‐tests among participants who received behavior therapy in the current sample showed no significant changes in neurocognitive measures from baseline to post‐treatment (all *p*s > .20).

**Table 2:** Descriptive statistics for childhood cognitive variables and follow‐up outcomes

Childhood cognitive variables	*N*	*M*	*SD*	Range
	[Post, baseline]			
WASI IQ	80	112	13	78–137
Stroop Color‐Word Test				
Color	79 [77, 2]	46.7	9.48	25–67
Word	80 [78, 2]	50.4	9.07	25–68
Color‐Word Interference	79 [77, 2]	51.8	8.41	22–70
Stop Signal Task				
Stop Signal Reaction Time	67 [59, 8]	254.4	59.6	114.0–452.2
Stop Accuracy	68 [63, 5]	55.1	8.93	25–70
Go Accuracy	69 [61, 8]	90.4	10.88	55.7–100
Change Signal Task				
Change Signal Reaction Time	67 [59, 8]	287.6	79.06	79.4–576.9
Change Accuracy	69 [63, 6]	50.3	12.45	17.5–75.0
Go Accuracy	70 [63, 7]	84.6	14.23	42.0–98.9
Auditory Consonant Trigrams				
Total Accuracy	80 [77, 3]	44.4	8.22	26–59
**Follow‐up outcomes**				
YGTSS Total Severity	80	16.23	9.54	0–38
YGTSS Impairment	80	10.00	10.77	0–40
GTS‐QOLI	72	20.62	17.83	0–82
QOLI T‐score	67	45.88	13.94	6–70

Total *N* = 80. For Childhood Cognitive Control variables, values in brackets indicate the number of participants with post‐treatment data (first value) and the number with baseline data carried forward (second value) due to missing post‐treatment data. GTS‐QOLI, Gilles de la Tourette Syndrome‐Quality of Life Scale; QOLI, Quality of Life Inventory; WASI, Wechsler Abbreviated Scale of Intelligence; YGTSS, Yale Global Tic Severity Scale.

An analysis of variance (ANOVA) found no significant differences in any neurocognitive variables in childhood between participants who completed the long‐term follow‐up (*n* = 80) and those who did not (*n* = 46). Next, analyses tested associations between childhood cognitive control and outcomes in three domains: tic severity and impairment, functional outcomes, and quality of life. Linear regression models tested associations with follow‐up YGTSS severity and impairment scores, and binomial logistic regression evaluated tic remission status (YGTSS Total Tic Score < 14). Functional outcomes were evaluated using binomial logistic regression predicting educational attainment (high school or less vs. postsecondary education or more), employment status (full‐time employed/student vs. part‐time employed/student or unemployed), and income (< $35,000 vs. ≥ $35,000). Quality of life was examined using linear regression predicting GTS‐QOL and QOLI scores; for interpretability, GTS‐QOL scores were reverse‐coded so that higher scores reflected better quality of life.

All participants, regardless of whether they had received behavioral therapy or psychoeducation in the original randomized trial, were included in the same analyses. Original treatment group was included as a covariate to adjust for any potential long‐term effects of initial intervention. All models also included childhood IQ (WASI) as a covariate to account for baseline cognitive ability. As some evidence suggests comorbid ADHD or OCD may exacerbate neurocognitive impairments in TS (Chang et al., 2018; Morand‐Beaulieu et al., 2025), all models also included a binary covariate indicating the presence of an ADHD and/or OCD diagnosis in childhood.^1^ For tic outcome models, post‐treatment YGTSS tic severity was co‐varied when predicting follow‐up YGTSS tic severity and remission status, and post‐treatment YGTSS impairment was co‐varied when predicting follow‐up YGTSS impairment.

## Results

Table 2 presents descriptive data for childhood cognitive control variables, TS outcomes, and quality of life.

### TS outcomes

Table 3 presents childhood cognitive control predictors of TS outcomes. Childhood Stroop performance significantly predicted tic outcomes in early adulthood. Lower tic severity at follow‐up was predicted by higher Stroop Word scores (β = −0.23, *p* = .048) and lower Stroop Interference scores in childhood (β = 0.34, *p* = .005) – reflecting faster processing speed and more efficient inhibition and set‐shifting, respectively. Stroop Color scores showed a similar but nonsignificant pattern (β = −0.19, *p* = .091). For tic‐related impairment, none of the Stroop indices reached significance, although higher Color scores predicted lower impairment at a trend level (β = −0.22, *p* = .097). Lower Stroop Interference scores also predicted partial/full tic remission (OR = 0.91, *p* = .017). No other cognitive control measures significantly predicted TS outcomes.

**Table 3:** Childhood cognitive control predictors of long‐term tic outcomes

Task	Predictor variable	YGTSS severity		YGTSS impairment		Remission	
		*β*	*p*	*β*	*p*	OR	*p*
Stroop Color‐Word Test	Color	−0.19	.091	−0.22	.097	1.04	.230
	Word	−0.23	.048*	−0.20	.136	1.04	.216
	Color‐Word Interference	0.34	.005**	0.08	.575	0.91	.017*
Stop Signal Task	Stop Signal Reaction Time	0.03	.121	0.01	.715	1.00	.653
	Stop Trial Accuracy	−0.03	.848	0.05	.701	1.01	.662
Change Signal Task	Change Signal Reaction Time	0.03	.759	0.12	.261	0.99	.801
	Change Trial Accuracy	0.17	.197	0.04	.769	0.95	.142
Auditory Consonant Trigrams	Total Accuracy	0.03	.121	0.01	.715	1.00	.653

Linear regression models were used for YGTSS severity and impairment; logistic regression was used for tic remission. All models included childhood IQ, original treatment group, and presence of childhood ADHD and/or OCD diagnosis (binary variable) as covariates. Models predicting YGTSS severity and remission included post‐treatment YGTSS severity as a covariate. Models predicting YGTSS impairment included post‐treatment YGTSS impairment as a covariate. Partial/Full Remission = YGTSS Total Tic Score ≥ 14 (0) versus <14 (1). OR, odds ratio; YGTSS, Yale Global Tic Severity Scale. *p < .05, **p < .01, ***p < .001.

### Functional outcomes

Table 4 presents childhood cognitive control predictors of functional outcomes. Odds of postsecondary education were predicted by greater accuracy on CST Change trials (OR = 1.08, *p* = .013) – reflecting better inhibition and response flexibility – and by higher ACT total scores (OR = 1.10, *p* = .029) – reflecting stronger working memory. No childhood cognitive control measures predicted employment status, although there was a trend for higher total ACT scores (OR = 1.07, *p* = .052). Higher ACT total scores also predicted greater odds of earning above $35,000 (OR = 1.11, *p* = .008). CST Change trial accuracy showed a nonsignificant trend toward predicting income (OR = 1.06, *p* = .087).

**Table 4:** Childhood cognitive control predictors of long‐term functional outcomes

Task	Predictor variable	Education		Employment		Income	
		OR	*p*	OR	*p*	OR	*p*
Stroop Color‐Word Test	Color	1.03	.363	1.02	.568	1.03	.358
	Word	1.03	.327	1.01	.799	1.03	.382
	Color‐Word Interference	1.00	.988	0.98	.614	1.04	.257
Stop Signal Task	Stop Signal Reaction Time	1.00	.924	1.00	.870	1.00	.885
	Stop Trial Accuracy	1.04	.215	1.01	.670	1.06	.218
Change Signal Task	Change Signal Reaction Time	1.00	.434	1.00	.414	1.00	.899
	Change Trial Accuracy	1.08	.013*	1.01	.814	1.06	.087
Auditory Consonant Trigrams	Total Accuracy	1.10	.029*	1.07	.052	1.11	.008**

Results from logistic regression models. All models included childhood IQ, original treatment group, and presence of childhood ADHD and/or OCD diagnosis (binary variable) as covariates. Education = high school or below (0) versus postsecondary or higher (1); Employment = full‐time employment or full‐time student (1) versus part‐time employment, part‐time student, or unemployed (0); Income = < $35,000 (0) versus ≥ $35,000 (1). OR, odds ratio. *p < .05, **p < .01, ***p < .001.

### Quality of life

Table 5 presents childhood cognitive control predictors of quality of life. Higher Stroop Color (β = 0.61, *p* = .009) and Word (β = 0.58, *p* = .016) scores and lower Stroop Interference scores (β = −0.62, *p* = .025) predicted better TS‐specific quality of life (GTS‐QOL). For the more general QOLI, higher Stroop Color (β = 0.42, *p* = .032) and Word (β = 0.44, *p* = .024) scores predicted better global quality of life. No other cognitive control variables were significant predictors of either quality of life measure.

**Table 5:** Childhood cognitive control predictors of quality of life outcomes

Task	Predictor variable	GTS‐QOLI		QOLI	
		β	*p*	β	*p*
Stroop Color‐Word Test	Color	0.61	.009**	0.42	.032*
	Word	0.58	.016*	0.44	.024*
	Color‐Word Interference	−0.62	.025*	−0.25	.273
Stop Signal Task	Stop Signal Reaction Time	−0.01	.816	−0.02	.557
	Stop Trial Accuracy	0.08	.733	−0.12	.550
Change Signal Task	Change Signal Reaction Time	0.02	.366	0.02	.356
	Change Trial Accuracy	0.09	.629	−0.10	.521
Auditory Consonant Trigrams	Total Accuracy	−0.04	.870	−0.09	.682

Results from linear regression models. All models included childhood IQ, original treatment group, and presence of childhood ADHD and/or OCD diagnosis (binary variable) as covariates. GTS‐QOLI, Gilles de la Tourette Syndrome‐Quality of Life Scale; QOLI, Quality of Life Inventory. *p < .05, **p < .01, ***p < .001.

## Discussion

This is the first longitudinal study to examine the relationship between childhood cognitive control and long‐term outcomes in TS. Specific domains of childhood cognitive control – processing speed, inhibition, set‐shifting, and working memory – predicted tic severity and impairment, educational achievement, income, and quality of life in early adulthood, controlling for childhood IQ and comorbid ADHD and OCD. Together, findings suggest that these childhood cognitive control processes may play an influential role in the clinical course, functional outcomes, and quality of life for individuals with TS – highlighting decreased childhood cognitive control as a potential target for intervention. However, several aspects warrant further consideration.

Regarding TS outcomes, greater processing speed, inhibition, and set‐shifting in childhood predicted lower tic severity and impairment in early adulthood. These aspects of cognitive control may help individuals with TS detect premonitory urges or early tic movements, enabling timely and effective use of tic management strategies – and support flexible implementation across contexts. Such strategies may include techniques learned in behavioral therapy (Essoe et al., 2021; McGuire, Sturm, et al., 2021b) and/or compensatory suppression developed naturalistically (Barber et al., 2024; Matsuda, Kono, Nonaka, Fujio, & Kano, 2016). Indeed, this may explain why prior studies found that childhood cognitive control predicted response to both behavior therapy (Chang et al., 2018; McGuire, Sturm, et al., 2021b) and nonbehavioral treatments (Chang et al., 2018). Collectively, these findings suggest that aspects of cognitive control related to inhibition and set‐shifting may function as prognostic factors for tic inhibition – contributing to reduced tic severity across development and into early adulthood.

With respect to functional outcomes, greater working memory, divided attention, and flexible responding in childhood were associated with higher educational attainment and income. Considering that most participants were in early adulthood and income was modestly restricted in range, the income finding should be interpreted with caution. Regarding educational attainment, however, results are more compelling: cognitive control can facilitate academic success by helping individuals resist distractions, manage competing demands, and shift behavior in response to changing circumstances (Ahmed et al., 2019; Miller et al., 2012). Specifically for youth with TS, these capacities may also help minimize the disruptive effect of tics in the classroom and/or facilitate tic inhibition while engaged in other tasks (Conelea & Woods, 2008). Strengthening working memory, divided attention, and flexible responding could set children with TS on developmental trajectories that foster persistence in postsecondary education.

Lastly, greater processing speed and inhibition/set‐shifting in childhood predicted better quality of life in early adulthood. These cognitive capacities support efficient information processing, impulse control, and flexible responding – abilities central to goal‐directed behavior across life domains. They may also enhance adaptive coping (Hendricks & Buchanan, 2016; Pruessner, Barnow, Holt, Joormann, & Schulze, 2020), enabling individuals to better manage both everyday stressors and the unique challenges of living with TS (Conelea, Busch, Catanzaro, & Budman, 2014; Jalenques et al., 2012; Wadman, Tischler, & Jackson, 2013). For instance, an adolescent with TS who has stronger cognitive control may not only have inherent abilities to suppress tics but also effectively shift attention between tic suppression and socialization with peers, which could contribute to greater confidence and well‐being (Matsuda et al., 2016; Merl, Barber, Pitts, & Woods, 2025). In contrast, an adolescent who has lower cognitive control may have greater difficulty with tic suppression and struggle to balance multiple attentional demands (Conelea & Woods, 2008; Conelea, Woods, & Brandt, 2011), which in turn could lead to feelings of frustration that undermine well‐being (Matsuda et al., 2016; Wadman et al., 2013).

### Implications

Taken together, these findings have clear clinical and research implications. Within the extensive neurocognitive battery used to characterize cognitive control in this study, performance on the Stroop task (capturing processing speed, inhibition, and set‐shifting) most consistently predicted long‐term outcomes. This brief measure could be feasibly integrated into clinical practice to inform families about potential challenges and guide treatment planning; for example, adapting behavioral therapy for tics to accommodate executive functioning needs (Greenberg et al., 2023) or advocating for school supports to reduce cognitive control demands and facilitate tic management (Koutsoklenis & Theodoridou, 2012).

Extending from this clinical perspective, processing speed, inhibition, and set‐shifting relate not only to tic severity but also to broader domains of functioning, underscoring the importance of cognitive control as a therapeutic target for youth with TS. Several interventions have been shown to improve cognitive control in youth, including physical exercise (Liang, Li, Wong, Sum, & Sit, 2021; Qiu, Liang, Wang, Zhang, & Shum, 2023; Zhou, Jiang, Wang, & Dou, 2025), working memory training, which yields reliable though domain‐specific gains (Qiu et al., 2023; Veloso, Vicente, & Filipe, 2020; Westwood et al., 2023; Zhou et al., 2025), and mindfulness‐based interventions (Ahmed Aboalola, 2024; Mak, Whittingham, Cunnington, & Boyd, 2018). Future research should test these approaches in youth with TS to determine whether they confer similar cognitive control improvements in this population. Longitudinal studies are also needed to clarify whether bolstering cognitive control in TS translates into long‐term benefits for clinical symptoms, functional adaptation, and quality of life.

### Limitations

Despite the numerous strengths of the study, several limitations warrant consideration. Participants were primarily male and White. While this reflects the typical demographic profile of TS samples (Bitsko et al., 2014; Xiong et al., 2024), it limits the generalizability of findings. Future work should examine whether similar neurocognitive predictors of long‐term outcomes are observed in more diverse patient groups. The study was also limited in its ability to examine socioeconomic influences on long‐term outcomes. Parental education and occupation were reported descriptively to provide context regarding participants' socioeconomic background, and most families were highly educated and professionally employed. An important future direction is exploring how variability in these and other socioeconomic factors may influence the observed associations.

Additionally, the present study did not directly examine emotional factors, such as anxiety or difficulties in emotion regulation, which are common among youth with TS and may interact with cognitive control processes in shaping long‐term clinical and functional trajectories. It is also possible that improvements in broader behavioral or emotional symptoms over time contributed to improved tic severity and functioning during the follow‐up period. Future research should examine how neurocognitive and emotional factors jointly influence long‐term outcomes in TS.

Some post‐treatment neurocognitive data were missing; although most could be carried forward from baseline performance, remaining missingness may have introduced bias. Neurocognitive data were also not collected beyond post‐treatment, preventing examination of change in cognitive control across the follow‐up period. Future studies in TS should include repeated neurocognitive assessments to clarify how developmental trajectories of cognitive control may shape long‐term outcomes.

Finally, the study cannot fully account for the multitude of extraneous variables and contextual influences, such as family support, environmental accommodations, and other interventions that may contribute to the outcomes observed at follow‐up.

## Conclusion

In summary, childhood cognitive control abilities, particularly processing speed, inhibition, set‐shifting, and working memory, predicted clinical, functional, and quality of life outcomes for young adults with TS. These findings highlight specific cognitive control processes as potential mechanisms underlying patient‐centered outcomes, underscoring the need for future research to develop and test interventions that can strengthen these abilities in children with TS. Ultimately, this line of work could improve long‐term life trajectories for individuals living with TS.

## Ethical considerations

Study procedures were approved by the University of California, Los Angeles Institutional Review Board (UCLA IRB; approval date: November 27, 2015; protocol number: IRB#15‐001512). All procedures were conducted in accordance with institutional and federal ethical standards. Written informed consent was obtained from all adult participants. For participants under 18 years of age, assent was obtained along with permission from a parent or legal guardian.

**Key points**

**What's known?**

- Tourette syndrome (TS) is associated with difficulties in cognitive control, including inhibition, working memory, and cognitive flexibility. However, the role of neurocognitive processes in predicting long‐term outcomes for TS has not been examined.

**What's new?**

- This study shows that stronger cognitive control in children with TS predicted better clinical, functional, and quality of life outcomes in early adulthood approximately 11 years later. Faster processing speed and more efficient inhibition and set‐shifting predicted lower tic severity and higher quality of life. Stronger working memory and response flexibility predicted higher educational attainment and income.

**What's relevant?**

- Childhood cognitive control may serve as a clinically meaningful prognostic factor and a potential intervention target to improve long‐term outcomes in TS.

## Data Availability

The data that support the findings of this study are available from the corresponding author upon reasonable request.

## Endnote

1 At baseline, 19 participants (24%) met diagnostic criteria for ADHD, 15 (19%) for OCD, and 4 (5%) for both disorders, resulting in 30 participants (38%) with at least one of these comorbidities.
